# Profiling Analysis of Volatile and Non-volatile Compounds in *Vitis Vinifera* Berries (cv. Chardonnay) and Spontaneous Bud Mutation

**DOI:** 10.3389/fnut.2021.715528

**Published:** 2021-08-06

**Authors:** Ting Zheng, Saihang Zhang, Xiangpeng Leng, Ehsan Sadeghnezhad, Teng Li, Tariq Pervaiz, Fanqi Liu, Haifeng Jia, Jinggui Fang

**Affiliations:** ^1^Key Laboratory of Genetics and Fruit Development, College of Horticulture, Nanjing Agricultural University, Nanjing, China; ^2^Institute of Grape Science and Engineering, College of Horticulture, Qingdao Agricultural University, Qingdao, China; ^3^Taiyihu International Winery Ecological and Cultural Zone, Weihai, China; ^4^China Wine Industry Technology Institute, Yinchuan, China

**Keywords:** bud mutation, chardonnay, grape, hormones, metabolome

## Abstract

A novel clonal variety of *Vitis vinifera* was identified from “Chardonnay” using inter-simple sequence repeat (ISSR) markers and called “bud mutation. ” The metabolomic profiles in Chardonnay and bud mutation berries indicated essential differences in the expression of key genes in the pathways of 2-C-methyl-D-erythritol-4-phosphate (MEP) and lipoxygenase-hydroperoxide lyase (LOX-HPL). Bud mutation fruits also matured 10 days earlier than Chardonnay and have higher carotenoid, sugar, and acidic compound contents. Furthermore, the gene expression was examined in the biosynthetic pathways of two ripening-associated hormones, abscisic acid (ABA) and jasmonic acid (JA), which significantly increased in bud mutation compared with the Chardonnay fruit. The synthesis and metabolism of amino acids, terpenes, fatty acids, volatile components, and specialized metabolites significantly increased in bud mutation. Therefore, in comparison with Chardonnay, bud mutation is considered a highly aroma-producing grape variety for an improvement in the beverage industry.

## Introduction

Grapes contain a variety of highly nutritive compounds, namely, sugars, organic acids, vitamins, amino acids, and crude fiber. In addition to these conventional nutrients, grapes are also rich in resveratrol and polyphenols (1). Aroma is not only an important indicator for evaluating the quality and commerciality of grapes and wines, but also an important factor that attracts consumers and enhances market competitiveness (2, 3). There are more than 1,300 kinds of volatile compounds in grapes and wines related to aroma, mainly including terpenoids and aromatic volatile aliphatic, pyrazine, and sulfur compounds (4, 5). Terpenoid metabolism is the main pathway of plant characteristic aroma synthesis (6). Terpenoids are precursors for the synthesis of main aroma substances in many plants. More than 70 kinds of terpenes have been identified in grapes, mostly monoterpenes, diterpenes, and sesquiterpenes (7). The mevalonate (MVA) pathway is mainly used to synthesize sesquiterpene and triterpene aroma components, while the methylerythritol phosphate (MEP) pathway is mainly used to synthesize monoterpenes, tetraterpenes, and diterpenes. The MVA and MEP pathways are not completely independent. MVA pathway synthesis products can enter plastids to form monoterpenes and diterpenes (8). Aromatic substances are one of the main factors that contribute to the quality of grape products, and are generated from biochemical reactions, microbial metabolism, and chemical or enzymatic reactions during wine storage (9).

Chardonnay, one of the most popular white grape varieties in the world, originated in Burgundy, France (10). Compared with other grape varieties, it has the advantages of early fruit maturity, high maturity, and high wood maturity. It was planted in Shandong, Shanxi, and Ningxia in China (11). Chardonnay contains some volatile compounds, such as diketones, acetates, fusel alcohols, volatile phenols, and lactones (12–14), but its fruit has no typical aroma, which is a major challenge in the production of high-quality white wine. Recently, a cultivar with fragrant flesh was found in a Chinese vineyard, and it possessed tissues similar to those of Chardonnay but had a superior aroma when processed at the Rushan Taiyihu Winery in Jiaodong. In vineyards, normal plants sometimes exhibit a common variation called a bud mutation (15). Due to stable clone propagation, a bud mutant that reveals the growth mechanism of a fruit variety is a valuable resource to breeders (15). Therefore, clonal varieties of Chardonnay could be developed to improve the quality and quantity of aroma chemicals during development and their adaptive attributes to the environment (16).

The objective of this study was to analyze the aroma components in the bud mutant grape and determine the key biosynthesis steps involved in the aroma formation process by systematically comparing the components and synthesis of hormone and metabolomics profiles of the aroma formation pathway in grapes. The results will be helpful for developing improved agronomic techniques to increase grape aroma content and reveal the regulatory pathway of grape aroma formation, and have a high potential in the generation of favorable resources for the breeding of new varieties.

## Materials and Methods

### Plant Material

The experiments were performed at the Lushan Vineyard in Shandong (37° 09′ N, 119° 10′ E). Vines were managed with standard cultivation techniques, such as pruning, yield requirements, and harvest standards. Four-year-old Chardonnay and its mutants were used as experimental material. Bud mutation emerged from one branch of the Chardonnay plants, and the fruits were enriched with muscat flavor. The original mutant grapes were selected from Chardonnay mutant buds and then cloned and propagated on a large scale through vegetative propagation.

The mutated grapevine branch was grown and used as experimental material. Ten plants were randomly selected from both cultivars, including cv. Chardonnay and cv. “Mutant.” Different parts of the grape plants, such as the fruit, branches, and leaves, were randomly collected at the time of maturation. After weighing, berries were peeled and separated into skin and pulp, frozen immediately in liquid nitrogen, and stored at −80°C for further analysis.

### Determination of Hormone and Sugar Content

The grape berries were cross-sectioned and longitudinally cut along the central axis into slices with a thickness of 5 mm. The cutting surface, fruit brush, and seed structure were observed under a stereomicroscope. The horizontal and vertical lengths of the berries were measured using an automatic vernier caliper. Total soluble solids (TSSs) were determined using a portable hand-held dialyzer (PAL-1, ATAGO, Tokyo, Japan), while sugar content was assayed according to the protocol described by Jia et al. (17). The determination of ABA, IAA, and JA content was performed using the method described by Jia et al. (18), and acidity was determined with the method described by Bissell et al. (19). The determination of amino acid content was performed using the method described by Zheng et al. (1), and aromatic precursor nitrogen (APN) was calculated from the concentrations of certain amino acids using the formula proposed by Valdes et al. (20).

### Determination of Carotenoid and Chlorophyll Contents

Fresh berries (0.5 g) were homogenized with 95% ethanol (10 ml), and the mixture was centrifuged (5,550 g) at 4°C for 15 min. The supernatant was separated, and 0.5 ml of the supernatant was mixed with 95% ethanol (4.5 ml). Chlorophyll a, chlorophyll b, and carotenoid contents were determined with a spectrophotometer at 664, 649, and 470 nm, respectively (21).

### Total RNA Extraction, cDNA Synthesis, and Gene Expression

Total RNA was isolated with an RNA isolation kit (QIAGEN GmbH, Hilden, Germany) according to the instructions of the manufacturer. For reverse transcription, 2 μg of total RNA was used in the PrimeScript™ RT Reagent Kit (Takara, Kyoto, Japan). Primers were designed with the Primer5 software (http://www.premierbiosoft.com/) (Supplementary Table 1). *VvActin* was used as an internal control. The qPCR reactions (20 μl) contained 10 μl SYBR Premix Ex^Taq^ (Takara, Japan), 2 μl cDNA, and.4 μl of 10 μM forward and reverse primers, with the preliminary step at 95°C for 30 s, followed by 40 cycles at 95°C for 5 s, and 55°C for 30 s. The mixture was placed in ABI7900 Sequence Detector (Applied Biosystem Inc., Hercules, CA, United States) to detect gene expression. The experiments were repeated three times.

### DNA Polymorphism and Genetic Characterization

The leaves (250 mg) were ground with liquid nitrogen and used for the extraction of genomic DNA according to the instructions of the manufacturer of Chemagic DNA Plant Kit (PerkinElmer, Waltham, United States). Loci were amplified *via* PCR using primer sets for different standard locus markers (Supplementary Table 1). The cycling profile was as follows: 95°C for 3 min; 32 cycles of 94°C for 20 s, 53°C annealing for 20 s, 72°C extension for 2.5 min, and final extension at 72°C for 5 min. After the reaction was completed, the samples were stored at 4°C for agarose gel detection. The PCR products were checked by polyacrylamide gel electrophoresis.

### Volatile Identification With Gas Chromatography–Mass Spectrometry

Pulp and skin (2 g) were ground in liquid nitrogen and mixed with 3 g NaCl and 2 μl octanol (81.8 mg/L) as the internal standard. GC–MS was performed according to Wu et al. (3), with some modifications.

The samples were equilibrated for 10 min at 50°C and then extracted for 30 min with fiber coating divinylbenzene/carboxen/polydimethylsiloxane (DVB/CAR/PDMS) with 50/30-μm thickness. The fibers were inserted immediately into the GC injection port for 3 min at 260°C in the splitless mode to release the volatile compounds.

The released compounds were separated with an Agilent 7890 GC system (Agilent Technologies, Santa Clara, CA, United States) using an HP-INNOWAX column (30 × 0.25 mm i.d., 0.25 μm film thickness; J&W Scientific Inc., Folsom, CA, United States), and then coupled with an Agilent 5975 MS instrument (Agilent Technologies, Santa Clara, CA, United States). The temperature ramp was 40°C for 5 min; then 2°C/min to 70°C for 2 min; 3°C/min to 120°C, then 5°C/min to 150°C, and finally 10°C/min to 220°C for 2 min. Helium was the carrier gas, and a 1-ml/min flow rate was used. The transfer line and ion source temperatures were 280 and 230°C, respectively. The ionization energy and scanning rates were 70 eV and 2.88 scan/s, respectively. The mass spectrometer adopted the electron ionization (EI) mode. The detection range of mass spectrometry was from 29 to 540 m/z (17). Under same chromatographic conditions, the C7-C27 n-alkane series of retention indices (RIs) was calculated. The RI and mass spectra of defined standards were used to identify the compounds. For unavailable reference standards, tentative identifications were made based on the standard NIST2011 library and compared with RI references in the literature.

### Metabolite Analysis With Ultra-Performance Liquid Chromatography Tandem Mass Spectrometry

The skin and pulp tissues (100 mg) were ground using liquid nitrogen and homogenized with methanol (V/V 80%) and formic acid (V/V 0.1%). The samples were put on ice for 5 min, and centrifuged at 10,000 × g and 4°C for 5 min. The supernatant was diluted with LC-MS-grade water to a final concentration of 53% methanol. Subsequently, the supernatant was transferred to a new Eppendorf tube and was centrifuged at 15,000 × g and 4°C for 10 min. Finally, the sample was analyzed with the LC-MS/MS system.

Liquid chromatography tandem mass spectrometry was performed with the Vanquish UPLC system (Thermo Fisher Scientific, Waltham, MA, United States) coupled with an Orbitrap Q Exactive series mass spectrometer (Thermo Fisher Scientific, Waltham, MA, United States). The samples were inserted into the Hyperil Gold column (100 × 2.1 mm, 1.9 μm) with a flow rate of 0.2 ml/min. The positive polarity mode eluents were (A) 0.1% formic acid in water and (B) methanol. The negative polarity mode eluents were (A, pH 9) 5 mM ammonium acetate and (B) methanol. The solvent gradient was set for 16 min as follows: 1.5 min for 2% B; 12 min for 2–100% B; 14 min for 100% B; 14.1 min for 100–2% B; and 17 min for 2% B. Spray voltage 3.2 kV in the positive/negative polarity mode was used in the Q Exactive series mass spectrometer, and the capillary temperature was 320°C. The independent variables were sheathed gas pressure (35 arb) and aux gas flow rate at 10 arb (22).

### Metabolite Classification and Functional Analysis

The Kyoto Encyclopedia of Genes and Genomes (KEGG) (http://www.genome.jp/kegg/), HMDB (http://www.hmdb.ca/), and Lipidmaps databases (http://www.lipidmaps.org/) were used for metabolite annotation. The metaX software was used to perform principal components analysis (PCA) and partial least squares discriminant analysis (PLS-DA). Statistical significance (*P*-value) was calculated by univariate analysis (*t*-test). VIP > 1 and *P*-value < 0.05 and fold change ≥ 2 or FC ≤ 0.5 were judged as differential metabolites. Based on log2(FC) and –log10(*P*-value), filter metabolites of interest were identified with Volcano plots (17, 23).

### Statistical Analyses

SAS 9.2 (Inc. Cary, NC, United States) was used for statistical analyses. Samples with differences were analyzed by Duncan's multiple comparison test at *p* < 0.05 and ANOVA.

## Results and Discussion

### Differences in Genetic Background and Physiological and Biochemical Indicators Between Chardonnay and Bud Mutation

To identify the similarity in the genetic backgrounds of Chardonnay and bud mutation, 38 primer pairs were used as inter-simple sequence repeat (ISSR) markers to assay genetic polymorphisms by PCR. As shown in Supplementary Figure 1, the genetic relationship between them was phenotypically analyzed according to the gel image. The results showed that the alleles of both grapes were identical, indicating that the genetic backgrounds of “Chardonnay” and “bud mutation” were very similar. Chardonnay is commonly cultivated worldwide as a white grape variety, but its volatile aromatic compounds categorize it as a low-aromatic grape variety. The differences between Chardonnay and bud mutation were investigated (Supplementary Figure 2A). The internode length and thickness of branches increased more in bud mutation compared with Chardonnay (Supplementary Figures 2C,D), although, Chardonnay had more branch clusters than bud mutation (Supplementary Figure 2B).

**Figure 1:**
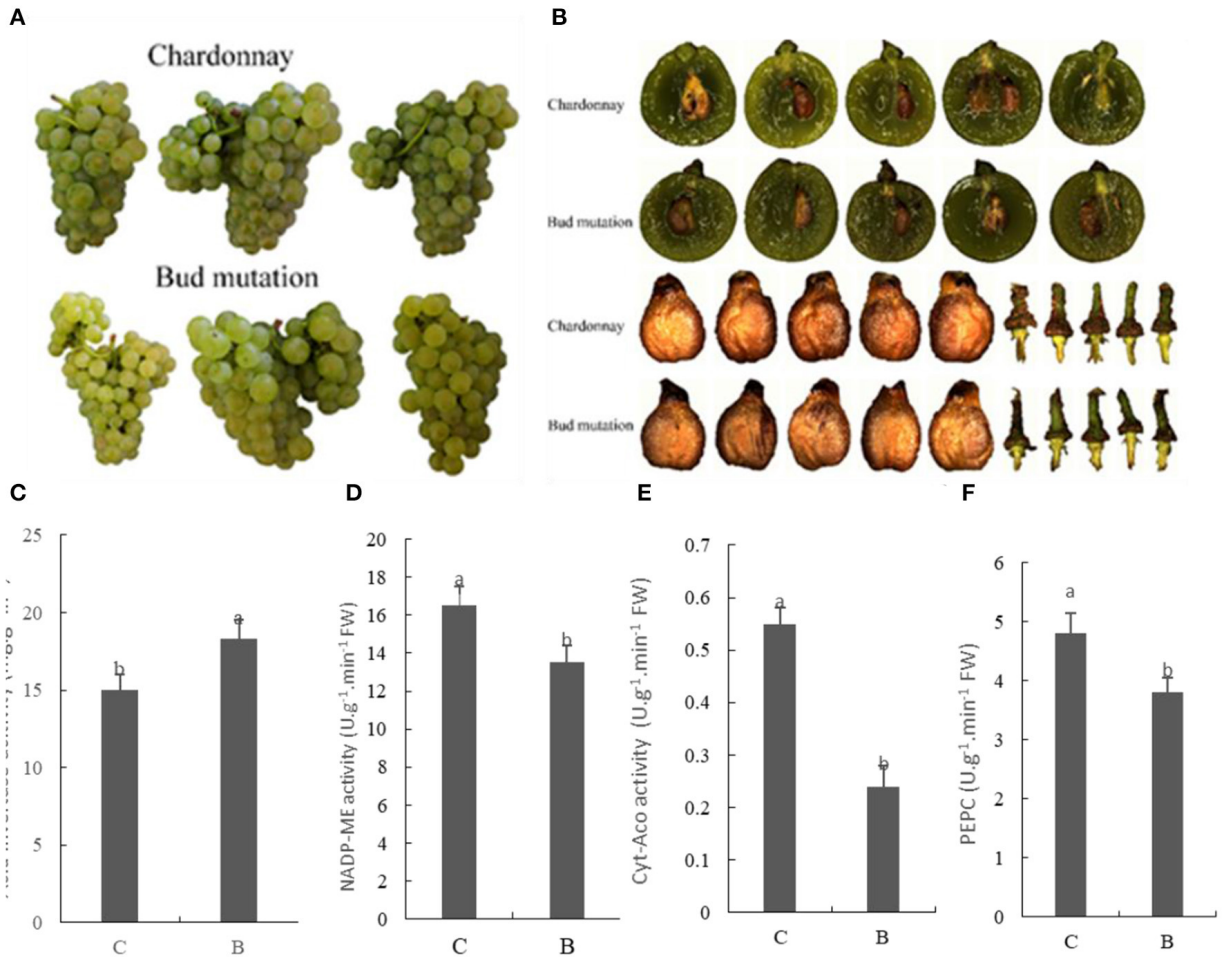
Fruit characterization and enzyme activities regarding sugar and acid metabolism in “Chardonnay” and “bud mutation” 90 days after flowering. **(A)** Fruit phenotype, **(B)** shape of seed and brush, **(C)** acid invertase, **(D)** NADP-malic enzyme (NADP-ME), **(E)** cytosolic aconitase (cyt-ACO), **(F)** phosphoenolpyruvate carboxylase (PEPC). Vertical bars represent the standard deviation (SD) of the mean (*n* = 3). Different letters indicate a significant difference at *p* < 0.05, as determined by Duncan's multiple range test. C, Chardonnay; B, bud mutation.

The results revealed that the number of seeds in bud mutation was higher, but the length of berries was the same in both varieties (Figures 1A,B, Table 1). The ear weight of bud mutation was less than that of Chardonnay, with no difference in seed weight. In the bud mutation fruit, the Brix and sugar content, namely, sucrose, glucose, and fructose, significantly increased compared with Chardonnay (Table 2). In many producing areas with relatively low calories in China, Chardonnay has a low glucose content, with which the wine produced has insufficient alcohol content. Sucrose needs to be added to increase the alcohol content (24). The sugar content of bud mutation is about 2.8 higher than that of Chardonnay, which can reduce or eliminate sucrose, increase the alcohol content appropriately, help to increase the fullness of the wine body, and thereby improve the quality of wine (25). Notably, the activity of some key enzymes involved in carbon fixation, such as NADP-malic enzyme (NADP-ME) and phosphoenolpyruvate carboxylase (PEPC), significantly decreased in bud mutation, while sugar-degrading enzymes, such as acid invertase, increased when compared with Chardonnay (Figures 1C,F). NADP-ME provides CO_2_ for fixation by RuBisCO (26), while PEPC adds bicarbonate (HCO${}_{3}^{-}$) to phosphoenolpyruvate (PEP) and forms oxaloacetate and inorganic phosphate (27). In addition, cytosolic aconitase (cyt-ACO), as a citrate-degrading enzyme, can change carbon flux with different metabolite biosynthesis pathways (28). The activity of these two enzymes was lower in bud mutation than in Chardonnay (Figures 1D,E).

**Table 1:** Agronomical parameters of “Chardonnay” and “bud mutation” at harvest (240 days after pruning).

**Agronomical parameters**	**Chardonnay**	**Bud mutation**
pH	3.9 ± 0.07^a^	4.2 ± 0.03^a^
°Brix	21.5 ± 0.15^b^	24.3 ± 0.27^a^
Total acidity (g L^−1^ tartaric acid)	4.89 ± 0.08^a^	5.12 ± 0.11^a^
Per fruit weight (g)	1.42 ± 0.03^a^	1.53 ± 0.07^a^
Clusters numbers (per plant)	13.5 ± 0.6^a^	12.8 ± 0.5^a^
Cluster weight (g)	133.5 ± 1.33^b^	142.7 ± 1.57^a^
Shoots numbers (per plant)	29.3 ± 0.4^a^	31.1 ± 0.8^a^
Shoot weight (kg per plant)	1.22 ± 0.13^a^	1.24 ± 0.09^a^
Fruit length horizontal (mm)	14.88 ± 0.11^a^	14.9 ± 0.08^a^
Fruit length vertical (mm)	15.07 ± 0.09^a^	14.95 ± 0.08^a^
Fruit brush length (mm)	3.5 ± 0.09^a^	3.7 ± 0.07^a^
Seed number (100 fruits)	156 ± 5.4^b^	187 ± 8.7^a^
Yield (kg/666.7 m^2^)	1022 ± 58.7^a^	1087 ± 98.7^a^
Germination (2019)	04/19	04/18
Flowering (2019)	05/25	05/19
Mature period (2019)	09/18	09/07
Germination (2020)	04/16	04/13
Flowering (2020)	05/15	05/11
Mature period (2020)	09/13	09/02

*Different letters indicate a significant difference at p < 0.05, as determined by Duncan's multiple range test*.

**Table 2:** Primary and secondary metabolites in juices belonging to the skins + pulp of “Chardonnay” and “bud mutation” at harvest (240 days after pruning).

**Metabolites**	**Chardonnay**	**Bud mutation**
**Sugars**		
Glucose (mg g^−1^)	41.3 ± 1.56^b^	47.2 ± 0.98^a^
Fructose (mg g^−1^)	48.9 ± 1.33^b^	70.1 ± 2.74^a^
Sucrose (mg g^−1^)	5.2 ± 0.11^b^	7.1 ± 0.17^a^
**Organic acids**		
Tartaric acid (mg g^−1^)	3.2 ± 0.07^b^	4.1 ± 0.11^a^
Malic (mg g^−1^)	0.9 ± 0.09^b^	1.5 ± 0.07^a^
Citric (mg g^−1^)	0.5 ± 0.04^a^	0.4 ± 0.03^a^
Oxalic (mg g^−1^)	1.1 ± 0.06^a^	1.3 ± 0.07^a^
Formic (mg g^−1^)	0.12 ± 0.02^b^	0.17 ± 0.02^a^
Propionic (mg g^−1^)	1.3 ± 0.05^a^	1.4 ± 0.07^a^
Ascorbic (mg g^−1^)	0.04 ± 0.02^a^	0.06 ± 0.02^a^
Shikimic (mg g^−1^)	0.07 ± 0.01^a^	0.06 ± 0.01^a^
**Amino acids**		
Alanine (mg L^−1^)	22.3 ± 0.53^a^	21.7 ± 0.86^a^
Arginine (mg L^−1^)	577 ± 15.33^b^	733 ± 13.97^a^
GABA (mg L^−1^)	8.9 ± 0.34^a^	10.5 ± 0.26^a^
Glutamine (mg L^−1^)	60.5 ± 3.34^a^	63.5 ± 2.98^a^
Isoleucine (mg L^−1^)	15.3 ± 0.46^b^	19.3 ± 0.34^a^
Leucine (mg L^−1^)	20.3 ± 0.23^a^	22.7 ± 0.79^a^
Proline (mg L^−1^)	15.6 ± 0.75^b^	25.7 ± 0.98^a^
Threonine (mg L^−1^)	13.5 ± 0.34^a^	15.5 ± 0.23^a^
Tyrosine (mg L^−1^)	6.9 ± 0.12^a^	6.6 ± 0.16^a^
Valine (mg L^−1^)	16.7 ± 0.72^b^	20.5 ± 0.56^a^
Aromatic precursor nitrogen (mg L^−1^)	72.7 ± 0.87^b^	84.6 ± 1.08^a^
**Phenolics**		
Epicatechin (mg L^−1^)	0.88 ± 0.05^a^	0.83 ± 0.04^a^
Syringic acid (mg L^−1^)	5.35 ± 0.23^a^	6.37 ± 0.45^a^
Chlorogenic acid (mg L^−1^)	40.6 ± 1.53^b^	51.22 ± 2.33^a^
Caffeic acid (mg L^−1^)	11.5 ± 0.87^a^	11.69 ± 0.45^a^
p-Coumaric acid (mg L^−1^)	12.7 ± 0.62^a^	11.77 ± 0.67^a^
Gallic acid (mg L^−1^)	12.1 ± 0.76^a^	13.45 ± 0.54^a^
*trans-*Caftaric acid (mg L^−1^)	403.5 ± 12.62^b^	456.32 ± 10.35^a^
**Polyamines**		
Ethanolamine (mg L^−1^)	8.55 ± 0.78^a^	10.37 ± 0.54^a^
Spermidine (mg L^−1^)	5.67 ± 0.66^b^	7.55 ± 0.45^a^
Putrescine (mg L^−1^)	2.45 ± 0.12^b^	3.03 ± 0.09^a^

*Different letters indicate a significant difference at p < 0.05, as determined by Duncan's multiple range test*.

The accumulation of tartaric acid and malic acid can also be considered one of the most essential features of the flavor of a wine (11). Meanwhile, bud mutation displayed high levels of tartaric, formic, and malic acids relative to Chardonnay, while citric, propionic, oxalic, ascorbic, and shikimic acid contents did not vary between these two varieties (Table 2). As an essential nitrogen source, amino acids support the growth of yeast during wine fermentation and affect fermentation kinetics (29, 30). The content of most kinds of amino acids increased in bud mutation, and arginine, valine, isoleucine, and proline significantly increased compared with Chardonnay. Among the phenolic compounds, chlorogenic acid and trans-caftaric acid significantly increased in bud mutation compared with Chardonnay (Table 2). Spermidine and putrescine, belonging to polyamines, were significantly increased in bud mutation (Table 2). The enhancement of these substances will make grapes and wines more flavorful.

### Aroma Profiling in Bud Mutation Is Different Than That in Chardonnay

**Figure 2:**
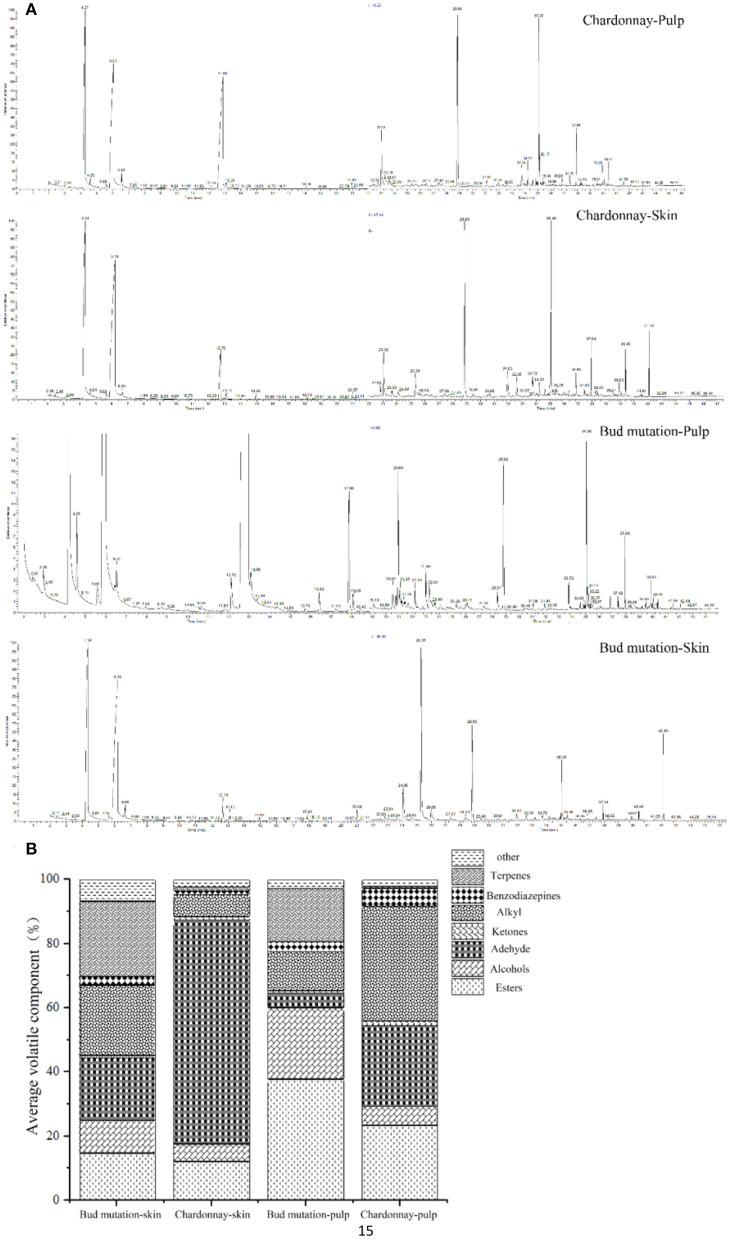
Base peak chromatogram (BPC) of **(A)** gas chromatography-mass spectrometry (GC-MS) and **(B)** rate of volatile components in the skin and pulp of “Chardonnay” and “bud mutation”.

To unravel the strong musky flavor of bud mutation, the aroma profiles of Chardonnay and bud mutation were determined (Figure 2A). A total of 301 volatile substances were detected in the bud mutation and Chardonnay fruits, and were divided into nine groups based on structure and chemical properties (Figure 2B). The volatile compounds were classified into different categories, namely, terpenes (31), esters (64), alcohols (28), aldehydes (22), ketones (28), alkanes (60), alkynes (6), acids (9), and benzodiazepines (32), in the skin and pulp of both cultivars (Supplementary Table 2). The proportion of volatile components in each sample differed among tissue types. For example, terpenes comprised 23.53 and 16.71% of the total volatile substances in the skin and pulp of bud mutation, respectively, while they decreased in the skin (0.09% of total) and pulp (0.06% of total) of Chardonnay. High levels of hexanal were only detected in Chardonnay pulp. The bud mutation skin contained higher concentrations of esters (3-hydroxymandelic acid, ethyl ester, di-TMS, acetate, and butyl ester), terpenes (geraniol, linalool, and D-limonene), alcohols (octanol, silanediol, dimethyl-, 2,6-octadien-1-ol, 3,7-dimethyl-, (Z)-, trans-2-hexenol, and 2-hexene-1-alcohol), aldehydes (phenylacetaldehyde, benzaldehyde, and 2-hexenal), and benzodiazepines (cyclopropane, propyl-, and octamethyl-). However, Chardonnay accumulated aldehydes (2,2-dimethyl-3-hydroxypropionaldehyde, phenylacetaldehyde, and hexanal), benzodiazepines (cyclopropane, propyl, and octamethyl), and trans β-ionone. This study revealed variations in the bud mutation and Chardonnay fruit ripening processes and aroma development (Supplementary Table 2). The odor activity value (OAV) of geraniol in wine is 30 μg/L, indicating that the geraniol in the peel of Chardonnay is lower than the minimum OAV. The OVA of linalool is 25 μg/L, indicating that the linalool in the peel of the bud mutation is above the OAV range, which can affect the composition of the volatile odor of wine (33). Furthermore, monoterpenes have a core role during the development of flavor that are like the grape itself and are also produced as an aromatic grape variety in ‘Muscat.’ Among the various monoterpenes in Muscat, several volatile compounds, namely, rose oxide, linalool, citral, geraniol, nerol, and citronellol, constitute the major components of the smell and taste of wine (31). In this study, geraniol content was 2.86 μg kg^−1^ FW in Chardonnay skin, while it was 237.71 μg kg^−1^ FW in bud mutation skin. No linalool was detected in the Chardonnay fruit, but it accumulated in the skin (56.26 μg kg^−1^ FW) and pulp (6.07 μg kg^−1^ FW) of bud mutation.

### Non-volatile Chemical Products Increased in Bud Mutation Fruit

LC-MS and PCA were performed on mature fruits to assess the gap in metabolomics profiling in bud mutation and Chardonnay. Metabolite scanning was performed on both positive and negative mode ions in the skin and pulp of both cultivars. Four hundred seventy-eight and 272 specialized metabolites were identified by the positive and negative modes, respectively, in the pulp and skin (Supplementary Table 3). To visualize the relative contribution of specialized metabolites in both cultivars, PCA was performed in two dimensions for the skin and pulp (Supplementary Figure 3). The PCA score plot showed that segregation of the sample replicates was evident between each other and among the respective components. The clonal variety or bud mutation described a higher percentage of data set variability in the positive and negative modes for skin (Supplementary Figures 3A,B; 74.78 and 64.74%, respectively) and pulp (Supplementary Figures 3C,D; 68.45 and 54.94%, respectively).

**Figure 3:**
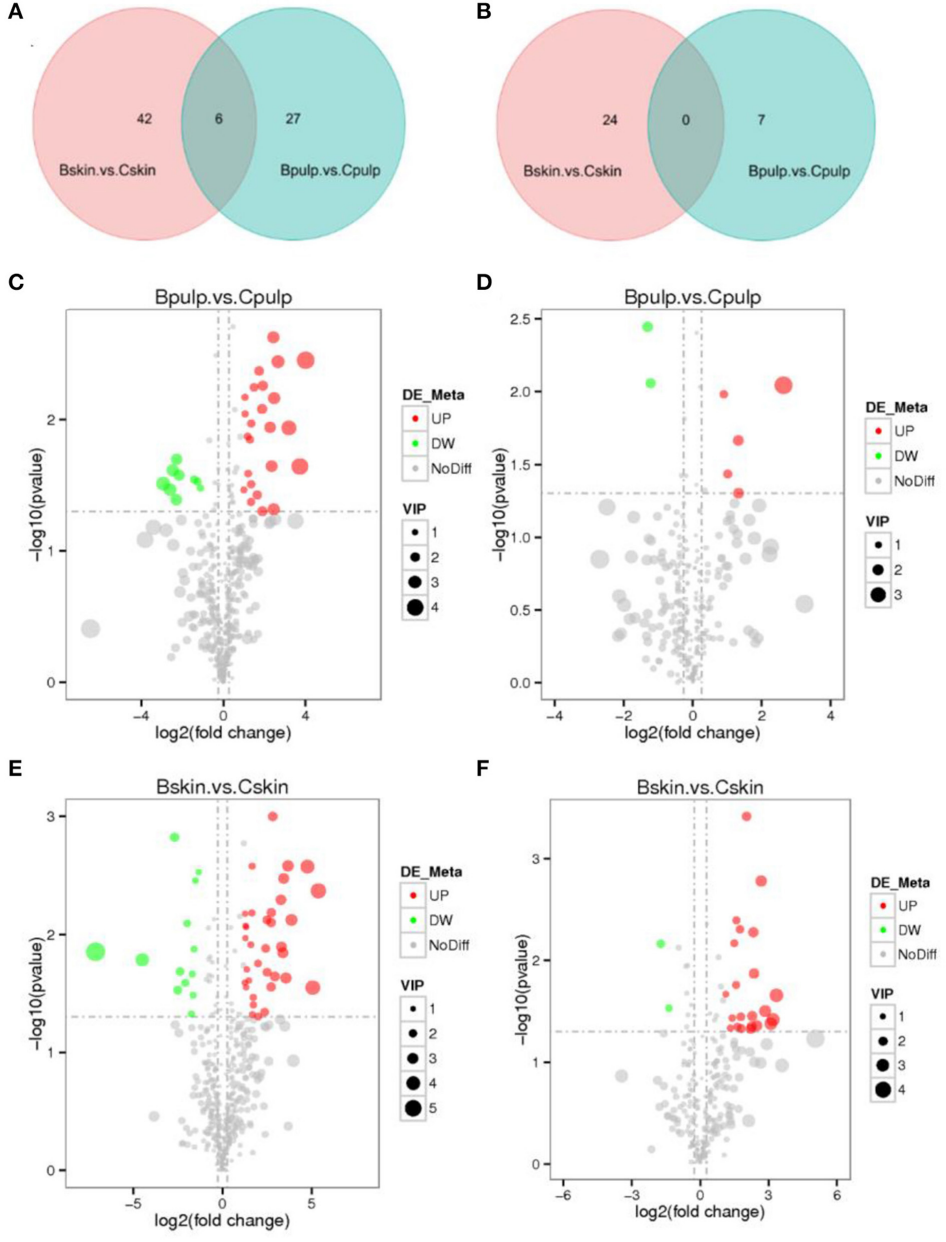
Venn diagram and changes in the identified metabolites in the skin and pulp of “Chardonnay” and “bud mutation.” The number of metabolites common to both platforms detected in the two ion modes, namely, **(A)** positive and **(B)** negative. The identity of individual metabolites that were measured on each platform is provided in Supplementary Table 4. Changes in up- and down-regulated important metabolites in **(C,D)** pulp and **(E,F)** skin using the two ion modes, namely, **(C,E)** positive and **(D,F)** negative.

The non-volatile substances in the skin and pulp were compared in both cultivars to identify compounds with different accumulation patterns. Non-volatile compounds were mainly revealed in the positive ion mode (Figures 3A,B, Supplementary Table 4). In the final data set, 75 (positive mode) and 31 (negative mode) distinct values of non-volatile compounds were identified in the pulp and skin. Six metabolites were quantified on more than one platform in the positive ion mode; however, no metabolites were recorded on common platforms in the negative mode.

Among the metabolites, 48 (35 up- and 13 down-regulated) and 24 (22 up- and 2 down-regulated) with different values that were significant in B-skin vs. C-skin in the positive and negative modes, respectively, were recorded (Supplementary Table 4). Furthermore, differences were observed in metabolite accumulation in the pulp, in which 33 (24 up- and nine down-regulated) and seven metabolites (five up- and two down-regulated) significantly changed in B-pulp vs. C-pulp in the positive and negative modes, respectively (Supplementary Table 4). In comparison with Chardonnay, the bud mutation fruit possessed a higher quantitative value of non-volatile substances in the pulp and skin. According to volcano plot analysis, the changes in non-volatile substances in both cultivars were identified (Figures 3C–F). In the positive ion mode, bud mutation skin accumulated more non-volatile components, such as cinchophen (log2FC = 3.65), geranic acid (log2FC = 2.73), citral (log2FC = 2), denin (log2FC = 2.94), and resveratrol (log2FC = 2.37), while some non-volatile chemical products underwent a substantial decline, such as 4-(5-propyl-2-pyridyl) benzonitrile (log2FC = −4.49) and methyl caffeate (log2FC = −7.11). In the negative ion mode, bud mutation skin accumulated enoic acid and ester compounds, such as cis-5,8,11,14,17-eicosapentaenoic acid (log2FC = 3.19) and propylparaben (log2FC = 3.09). In the bud mutation pulp, citra (log2FC = 12.9) content was higher, while acidic compounds, namely, 9-oxo-10(E), 12(E)-octadecadienoic acid (log2FC = −1.6), α-linolenic acid (log2FC = −2.16), and 4,5-dicaffeoylquinic acid (log2FC = −1.43), were significantly decreased in the positive ion mode.

### Enriched KEGG Metabolic Pathways in Bud Mutation and Chardonnay Fruits

**Figure 4:**
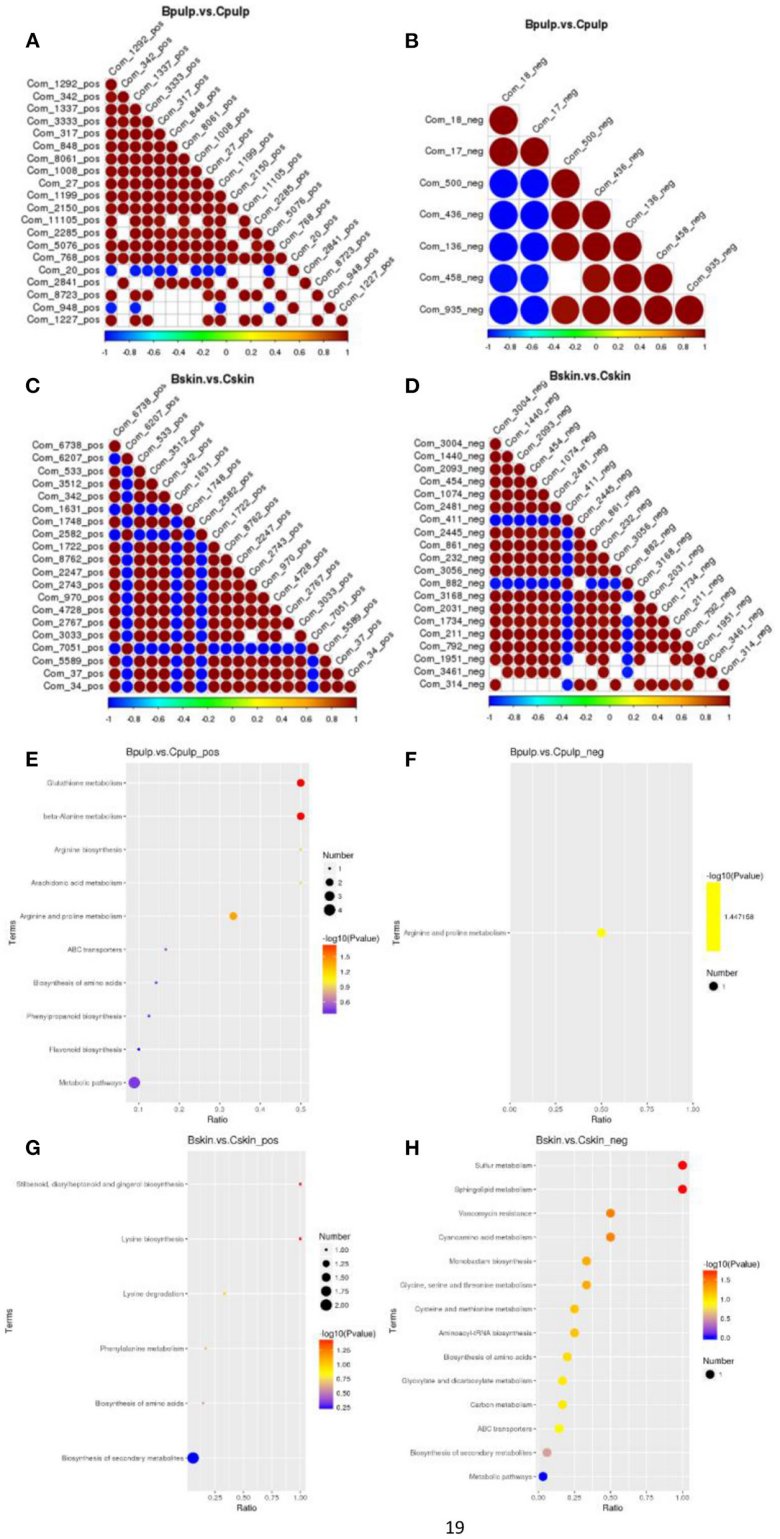
**(A–D)** Correlation analysis and **(E–H)** KEGG enrichment regarding metabolites in “Chardonnay” and “bud mutation.” Metabolites were identified using the positive mode in bud mutation vs. Chardonnay in **(A,E)** pulp and **(C,G)** skin. Metabolites were identified using the negative mode in bud mutation vs. Chardonnay in **(B,F)** pulp and **(D,H)** skin.

The correlation between different metabolites showed the changing trend in metabolites in the skins of Chardonnay and bud mutation in the cation mode, which had a positive correlation (0.93) (Figures 4A–D). The variations between the samples were observed predominantly in the synthesis and metabolism of amino acids and secondary metabolites from enriched KEGG metabolic pathways (Figures 4E–H, Supplementary Table 5). In the skins of bud mutation and Chardonnay, when metabolites were detected by the positive ion mode, metabolic pathways, such as stilbenoid, diarylheptanoid, and gingerol biosynthesis (map00945), and the biosynthesis of secondary metabolites (map01110) were different; however, different pathways, such as biosynthesis of amino acids (map01230), and glycine, serine, and threonine metabolism (map00260) were significantly enriched with differentially expressed genes (DEGs). Although, there were few differentially enriched pathways for pulp in the negative mode, metabolic pathways, such as phenylpropanoid biosynthesis (map00360), flavonoid biosynthesis (map00941), and arachidonic acid metabolism (map00590), were enriched in the positive mode (Figures 4E,F).

### Functional Annotation of Specialized Metabolites

**Figure 5:**
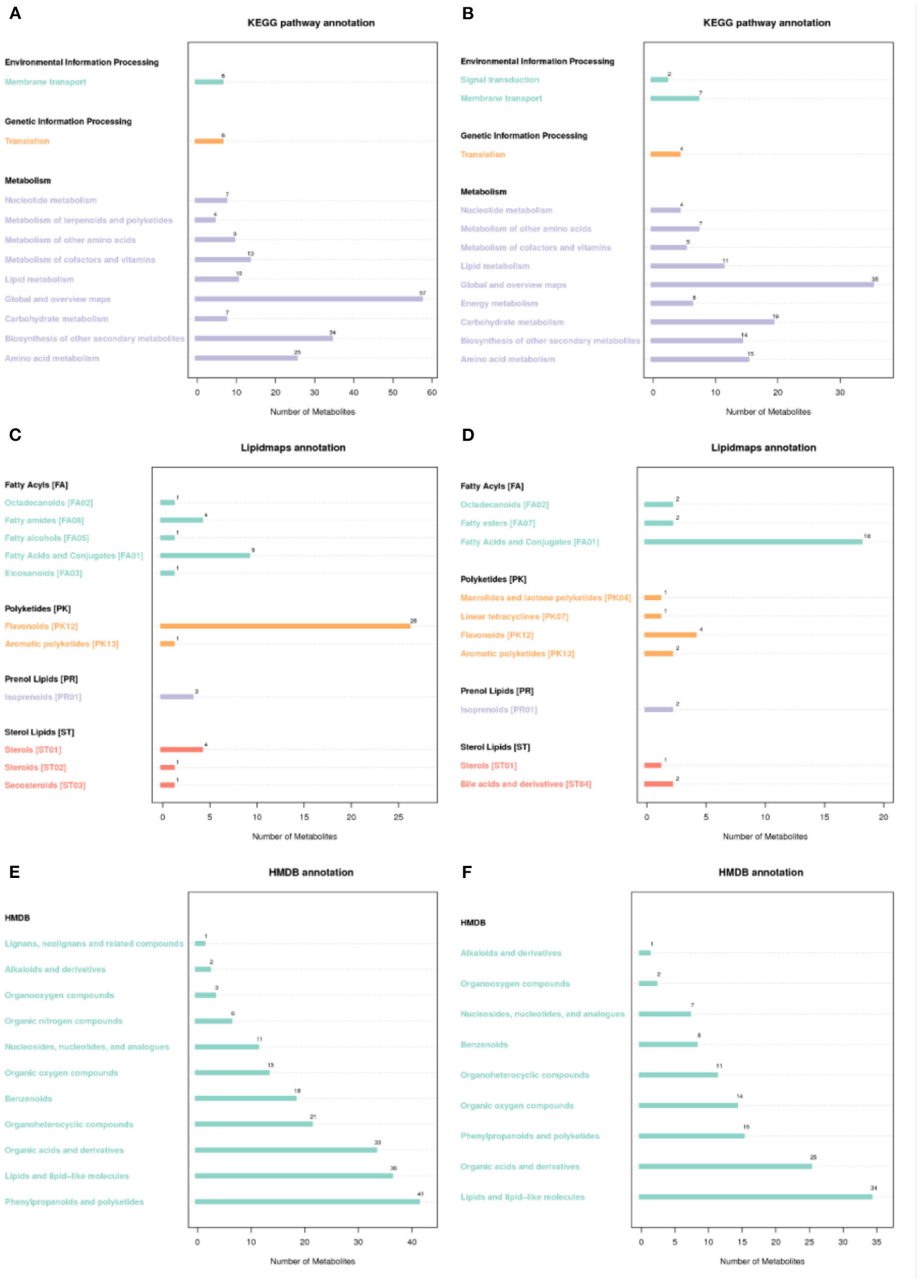
Annotation of metabolites using the **(A,B)** Kyoto Encyclopedia of Genes and Genomes (KEGG) pathway, **(C,D)** Lipidmaps, and **(E,F)** HMDB databases in “Chardonnay” and “bud mutation.” Metabolite scanning was performed in both the **(A,C,E)** positive and **(B,D,F)** negative ion modes.

All metabolites were annotated in KEGG pathways and classified into three major groups: environment information processing, genetic information processing, and metabolism (Figures 5A,B). Among the functional pathways, environment information processing was summarized into two major subgroups: signal transduction (2) and membrane transport (7) in the negative mode, while membrane transport (6) was only detected in the positive mode. Translation had the highest frequency of cohesive pathways among genetic information processing in the positive (6) and negative modes (4). Functional subgroups implicated in metabolic pathways, such as nucleotides, terpenoids and polyketides, amino acids, sugars, cofactors and vitamins, lipids, global and summary maps, and the positive and negative biosynthesis of secondary metabolites of various values, were also identified (Supplementary Table 6). Among metabolite annotation tools, the Lipidmaps (34) and HMDB (35) databases, which are devoted to metabolomics, were used (Figures 5C–F). According to Lipidmaps annotation, the major functional groups consisted of fatty acyls, polyketides, prenol lipids, and sterol lipids (Figures 5C,D). The subgroups that belonged to fatty acyls in the positive mode were octadecanoids, fatty amides, fatty alcohols, eicosanoids, fatty acids, and conjugates, while octadecanoids, fatty esters, fatty acids, and conjugates were found in the negative mode. Meanwhile, polyketides, such as flavonoids and aromatic polyketides, were found in the positive mode, while the subgroups in the negative mode contained macrolides and lactone polyketides, linear tetracyclines, flavonoids, and aromatic polyketides. Sterol lipids included sterols (ST01), steroids (ST02), and secosteroids (ST3) in the positive mode, while sterols (ST01) and bile acids and derivatives (ST4) formed subgroups in the negative mode (Supplementary Table 6). Furthermore, metabolite entries by HMDB annotated 11 and nine structures and pathways in the positive and negative modes, respectively (Figures 5E,F). Metabolomics analysis showed that the content of flavonoids and aromatic polyketones, such as cinchophen, geranic acid, and citral, significantly increased in bud mutation fruit compared with Chardonnay. Among them, citral had a higher content in the skin and pulp of bud mutation, which could improve the scent of bud mutation in a manner similar to Muscat grape.

### Changes in Endogenous Hormones Between Bud Mutation and Chardonnay

**Figure 6:**
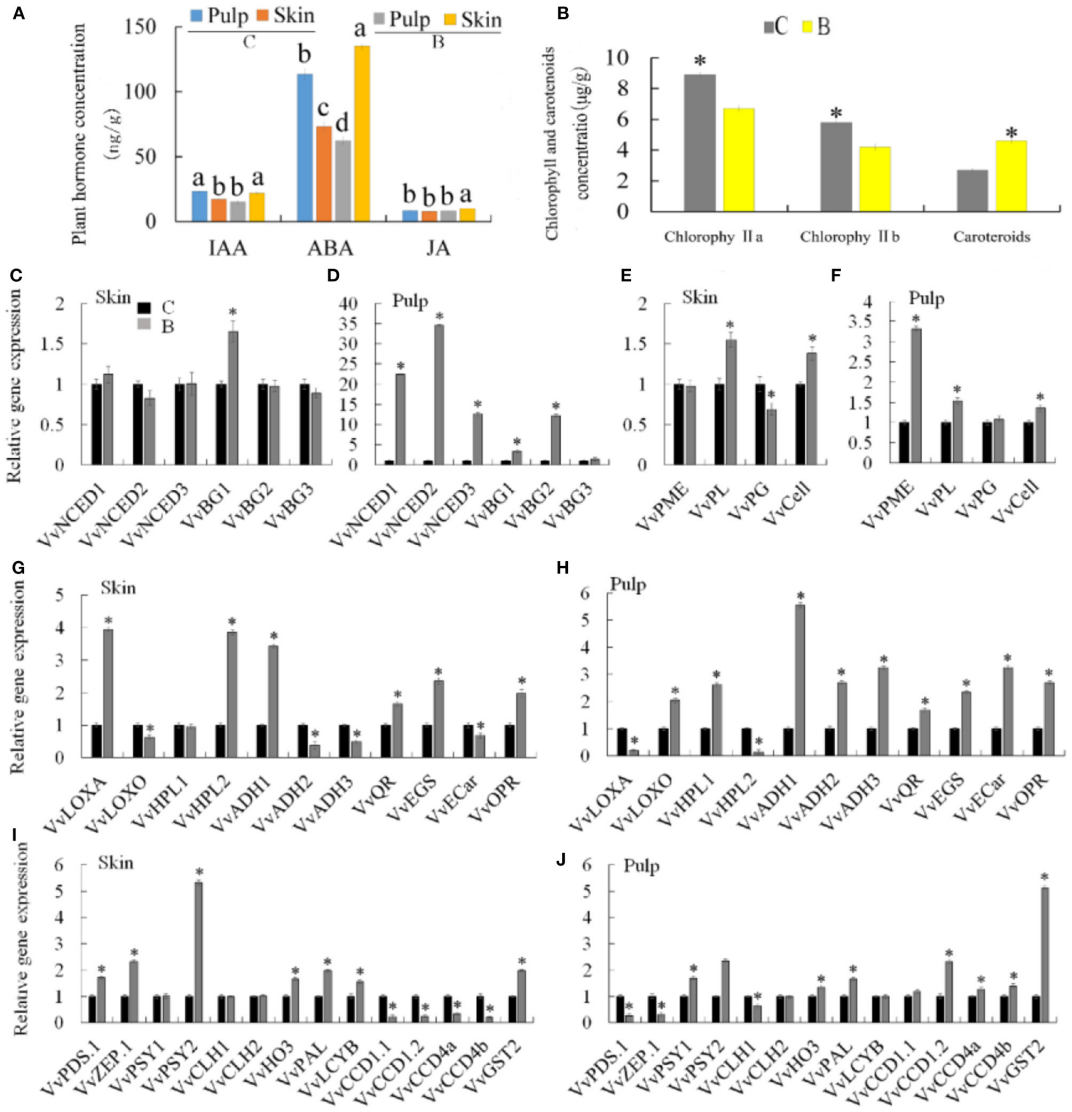
Changes in metabolism genes and content of chlorophylls, carotenoids, and hormones in “Chardonnay” and “bud mutation.” **(A)** Plant hormone concentration, **(B)** chlorophyll and carotenoid concentration. Gene expression involved in the pathway of **(C,D)** abscisic acid (ABA) biosynthesis, **(E,F)** fruit softening, **(G,H)** jasmonic acid (JA) biosynthesis, **(I,J)** carotenoid metabolism. ^*^Indicates the significance level in *P* ≤ 0.05. Different letters indicate a significant difference at *p* ≤ 0.05, as determined by Duncan's multiple range test. C, Chardonnay; B, bud mutation.

To understand whether the difference between bud mutation and Chardonnay metabolism was related to changes in endogenous hormones, hormones such as JA, ABA, and IAA, were detected in the skin and pulp of both varieties. ABA was significantly increased in the skin (135.14 ng/g.FW) of bud mutation compared with Chardonnay (112.6 ng/g.FW), while IAA did not change in the skin or pulp in either variety (Figure 6A). The expression levels of ABA biosynthetic genes in pulp, namely, *VvNCED1, VvNCED2, VvNCED3, VvBG1*, and *VvBG2*, significantly increased in the bud mutation cultivar, while most of the genes did not change in the skin, except *VvBG1*, which significantly increased compared with Chardonnay (Figures 6C,D). The accumulation of ABA may be the reason for early ripening in the bud mutation fruit. In addition, changes in the ABA content also affect the firmness of the fruit. For example, compared with Chardonnay, the firmness of the bud mutation fruit was reduced, and gene expressions associated with fruit softening, such as *VvPME, VvPL*, and *VvCell*, in the pulp significantly increased, while *VvPL* and *VvCell* were expressed more in the skin (Figures 6E,F). These results indicated that the bud mutation cultivar synthesized ABA more to promote fruit softening and accelerated the fruit ripening process.

### Pathway Analysis in the Production of Volatile Substances

In addition to monoterpene substances, other compounds, namely, C6 compounds (aldehydes, alcohols, lipids), linear volatile compounds, linear aliphatic alcohols, aldehydes, ketones, and esters, were detected in bud mutation, which were derived from fatty acid oxidative degradation (36). The methylerythritol phosphate (MEP) pathway and the lipoxygenase-hydroperoxide lyase (LOX-HPL) pathway are involved in the production of volatile substances in Muscat grapes (3). Therefore, the content of JA was assayed in the fruit and skin of bud mutation as a product of the LOX-HPL pathway, and was found to be significantly increased compared with Chardonnay (Figure 6A). The LOX-HPL pathway produces C6-compounds through the oxidative cleavage of polyunsaturated fatty acids (PUFAs), especially linoleic acid and linolenic acid, which accumulate during the fruit ripening process (37, 38). In addition, the correlation analysis showed that genes *VvLOXA* and *VvHPL2* were associated with the formation of hexenal and trans-2-pentanal during the synthesis of aroma substances in grapes (39). The pulps of the bud mutation fruit had more chemical compounds, such as nine, seven, and two more lipid, alcohol, and aldehyde compounds, respectively, when compared with the Chardonnay pulp. The pulp of the mutant bud fruit contained more chemical components, such as lipid, alcohol, and aldehyde compounds. Among them, the positive contributions to the volatile aroma included trans-geranic acid methyl ester and2-hexyn-1-ol, which belong to esters and alcohols, respectively (40, 41). In the bud mutation skin, two more alcohol, and one more aldehyde compounds were observed when compared with Chardonnay. By analyzing the expression levels in the LOX-HPL pathway, the expression levels of *VvLOXO, VvHPL1, VvADH1, VvADH2*, and *VvADH3* in the bud mutation pulp were significantly higher than that in Chardonnay (Figures 6G,H). In addition, the expression levels of *VvLOXA, VvHPL2*, and *VvADH1* in the bud mutation skin were higher than that in Chardonnay (Figures 6G,H).

Corresponding to the high expression of *VvLOXA* in the bud mutation skin, a high content of 2-hexenal was detected in the bud mutation skin, but trans-2-valeraldehyde was not detected in the skin. Exogenous ABA and JA can significantly increase the C6 aroma of wine grapes (42). These results suggest that aroma formation in the pulp and skin is affected by differential gene expression.

Furthermore, chlorophyll a and b contents significantly decreased in the bud mutation skin, while there was a significant increase in carotenoids when compared with Chardonnay (Figure 6B). Carotenoids belong to a group of isoprenoid molecules, synthesized by the MEP pathway, which can produce volatile substances in plants (43–45). In the MEP pathway, 1-deoxy-D-xylulose-5-phosphate synthase (DXS), 1-hydroxy-2-methyl-2-(E)-butenyl 4-diphosphate reductase (HDR), isopentenyl diphosphate isomerase (IDI), and geranyl pyrophosphate synthase (GPPS) play an important role, while phytoene synthase (PSY), zeaxanthin cyclooxygenase (ZEP), neoxanthine synthase (NXS), and 9-cis-epoxy carotenoid dioxygenase (NCED) are essential for the synthesis of carotenoids (6, 45–47).

In the study, the expression of *PSYs* in the skin and pulp of bud mutation was significantly higher than that in Chardonnay (Figures 6I,J). We identified four members of the *VvCCD* family (*VvCCD1.1, VvCCD1.2, VvCCD4a*, and *VvCCD4b*) in grapes. The studies on *VvCCD*s in grape and tomato fruits have found that the expression of this gene contributes to the formation of flavor volatile β-ionone (45, 48). The quantitative expression of genes and the detection results of volatile substances can explain the content of trans-beta-ionone in Chardonnay fruits and peels. GGPP is a common precursor for all plastid isoprenoids. It is produced by geranyl diphosphate synthase (GGPPS), and it catalyzes the condensation of one DMAPP unit and three IPPs. Lycopene is desaturated through the participation of lycopene desaturase (PDS), and then lycopene β-cyclase (LCYB) catalyzes the formation of γ-carotene from lycopene. *VvPDS1* was highly expressed in the bud mutation skin but decreased in the pulp. The expression level of two *VvGGPPS* genes in the skin of bud mutation was lower than that in Chardonnay, but increased in the pulp. The expression of *VvLCYB* in the bud mutation skin was significantly higher than that in Chardonnay. Therefore, key genes in the carotenoid biosynthesis pathway are more highly expressed in the bud mutation fruit, leading to higher carotenoid accumulation compared with Chardonnay (Figures 6I,J).

### Production of Terpenoids Increased in Bud Mutation Fruit

The most volatile compounds in grapes and wines are extracted from terpenes (monoterpenes, sesquiterpenes), fatty acid derivatives, sulfides, and methoxypyrazine (49, 50), and acetyl-CoA is the precursor to the synthesis of terpene compounds (8). Isoprenoids are one of the main classes of plant secondary metabolites, commonly known as terpenoids and terpenes, and include quinones, sterols, polyterpene alcohols, chlorophyll, carotenoids, and phytohormones (ABA, gibberellin, cytokinin, and brassinolide) (2, 51). Isoprenoids contain a low olfactory threshold and, therefore, have a higher sensory effect on grape aroma (52). These ketones have a very low perception threshold, 0.09 μg/L (33). In grapes and wines, a total of 22 different monoterpenes have been found, half of which are linalool derivatives (32), suggesting that linalool is an essential monoterpene in wine. Therefore, similar to Muscat, geraniol, linalool, and other monoterpenes were also found in the bud mutation fruit. Linalool also accumulates in some non-Muscat aroma varieties during the early stages of fruit development, but its concentration rapidly decreases when the berries mature (33, 37). In contrast, among Muscat aroma varieties, linalool increases rapidly when the color of fruit changes (53). The MEP and LOX-HPL pathways increase volatile substances in Muscat grapes (3), and lipoxygenase (LOX), hydrogenase (HPL), and alcohol dehydrogenase (ADH) are the key enzymes in these pathways (54). The expression of four key genes in the MEP pathway and five terpene biosynthesis genes for monoterpene biosynthesis was analyzed in grape berries (Supplementary Figure 4). The expression levels of the main MEP pathway enzymes, namely, *VvDXS, VvDXR*, and *VvGPPS*, were higher in the bud mutation fruit in the skin and pulp than in the Chardonnay mutation fruit. (Supplementary Figures 4A,B). The first step of the MEP pathway and carotenoid biosynthesis was catalyzed by deoxy-D-xylulose-5-phosphate synthase (DXS). The *VvDXS* gene was found to be responsible for the characteristics of Muscat by QTL analysis, which could explain 17–93% of the change in linalool, nerol, and geraniol compounds (55, 56). The expression pattern of the *VvIDI* gene was significantly increased in the bud mutation pulp but not in the skin in comparison with Chardonnay. Furthermore, the expression of *VvTPS24* and *VvTPS56* in the skin and *VvTPS58* in the bud mutation pulp indicated a significant increase. Geranyl pyrophosphate synthase (*VvGPPS*) is a key gene in the MEP pathway, and it catalyzes the production of geranyl pyrophosphate as a substrate for monoterpene synthase (47). In Pinot Noir, *VvTPS56* and *VvTPS58* were determined to be linked to (3S)-Linalool/(E)-Nerolidol synthase and linalool/(E)-nerolidol/(E,E)-geranyl linalool synthase, respectively, and their overexpression in the skin of the bud mutant suggested the accumulation of monoterpenes (46). Therefore, the expression of terpene synthase-related genes in the bud mutant fruit significantly increased than that in Chardonnay.

## Conclusion

A clonal variety of Chardonnay, called bud mutation, was identified, which could mature 10 days earlier than the Chardonnay fruit, and was enriched in linalool, geraniol, acetic acid, butyl ester, and other components of the Muscat fragrance. The production of volatile and non-volatile compounds in the bud mutation fruit was increased by the expression of key enzymes in the MEP and LOX-HPL pathways, which were significantly higher than that of Chardonnay. Furthermore, carotenoid, sugar, tartaric acid, and malic acid contents accumulated more in bud mutation than in Chardonnay. Although, Chardonnay is widely cultivated for wine production relative to other grape varieties, the lack of a typical rich aroma in the fruit is a major challenge for brewing Chardonnay into a high-quality white wine. Therefore, clonal varieties of Chardonnay have a high potential for the preservation of the quality and quantity of aromatic compounds for the beverage industry.

## Data Availability Statement

The original contributions presented in the study are included in the article/Supplementary Material, further inquiries can be directed to the corresponding authors.

## Author Contributions

TZ and SZ: writing—original draft, conceptualization, validation, formal analysis, and data curation. XL, TL, and FL: methodology, software, formal analysis, investigation, resources, and visualization. ES and TP: writing, review, and editing. HJ: funding acquisition, project administration, writing, review, and editing. JF: writing—review and editing. All authors contributed to the article and approved the submitted version.

## Conflict of Interest

The authors declare that the research was conducted in the absence of any commercial or financial relationships that could be construed as a potential conflict of interest.

## Publisher's Note

All claims expressed in this article are solely those of the authors and do not necessarily represent those of their affiliated organizations, or those of the publisher, the editors and the reviewers. Any product that may be evaluated in this article, or claim that may be made by its manufacturer, is not guaranteed or endorsed by the publisher.

## Figures and Tables

**Table:** 

ABA,	Abscisic acid
ADH,	Alcohol dehydrogenase
BG,	Beta- glucosidase
CCD,	Carotenoid cleavage dioxygenase
CLH,	Chlorophyllase
DAD,	Diode array detector
DMAPP,	Dimethylallyl diphosphate
DXR,	1-deoxy-D-xylulose-5-phosphate reductoisomerase
DXS,	1-Deoxy-D-xylulose-5-phosphate synthase
ECar,	(E)-beta-caryophyllene synthase
EGS,	Eugenol synthase
FLD,	Fluorescence detector
GC-MS,	Gas chromatography-mass spectrometry
GGPPS,	Geranylgeranyl diphosphate synthase
GPPS,	Geranyl pyrophosphate synthase
GST,	Glutathione S-transferase
HDR,	1-hydroxy-2-methyl-2-(E)-butenyl 4-diphosphate reductase
HMDB,	Human Metabolome Database
HO,	Heme oxygenase
HPLC,	High-performance liquid chromatographic
IAA,	Indole-3-acetic acid
IDI,	Isopentenyl diphosphate isomerase
IPP,	Isopentenyl diphosphate
JA,	Jasmonic acid
KEGG,	Kyoto Encyclopedia of Genes and Genomes
LCYB,	Lycopene ε-cyclase
log2FC,	Log2 fold-change
LOX-HPL,	Lipoxygenase-hydroperoxide lyase
MEP,	2-C-methyl-D-erythritol 4-phosphat
NCED,	9-cis-epoxycarotenoiddioxygenase
NXS,	Neoxanthin synthase
OPR,	12-oxophytodienoate reductase
PAL,	Phenylalanine ammonia-lyase
PDS,	Participation of lycopene desaturase
PDS,	Phytoene desaturase
PG,	Polygalacturonase
PL,	Pectate lyase
PME,	Pectin methylesterase
PSY,	Phytoene synthase
PUFA,	Polyunsaturated fatty acids
qPCR,	Quantitative real-time PCR
QTLs,	Qualified Teacher Learning and Skills
QR,	Quinine Oxidoreductase
TA,	Titratable Acid
TPS,	Terpene Synthase
TSS,	Total Soluble Solids
ZEP,	Zeaxanthin Epoxidase
