# Supplementary material for: Profiling Analysis of Volatile and Non-volatile Compounds in Vitis Vinifera Berries (cv. Chardonnay) and Spontaneous Bud Mutation
Source: Front Nutr. 2021 Aug 6;8:715528. doi: 10.3389/fnut.2021.715528 (PMC8378792; doi:10.3389/fnut.2021.715528)
Supplement: Supplementary file 7 [file Data_Sheet_1.docx]

SUPPORTING INFORMATION

Profiling analysis of volatile and non-volatile compounds in *Vitis vinifera* berries (cv. Chardonnay) and spontaneous bud mutation

Saihang Zhang^1#^, Ting Zheng^1#^, Xiangpeng Leng^2#^, Ehsan Sadeghnezhad^1^, Teng Li^1^, Tariq Pervaiz^1^, Fanqi Liu^3^, Haifeng Jia^1,4*^, Jinggui Fang^1,4*^

**Author Affiliations**

1. Key Laboratory of Genetics and Fruit Development, College of Horticulture, Nanjing Agricultural University, 1stWeigang Rd., Nanjing 210095, China

2. Qingdao Agricultural University, Qingdao, Shandong Province

3. Taiyihu international winery ecological and cultural zone, Rushan Weihai City, Shandong Province

4. China Wine Industry Technology Institute

***Corresponding author：Haifeng Jia, Jinggui Fang**

**Email:** [**jiahaifeng@njau.edu.cn**](mailto:jiahaifeng@njau.edu.cn)**,** [**fanggg@njau.edu.cn**](mailto:fanggg@njau.edu.cn)


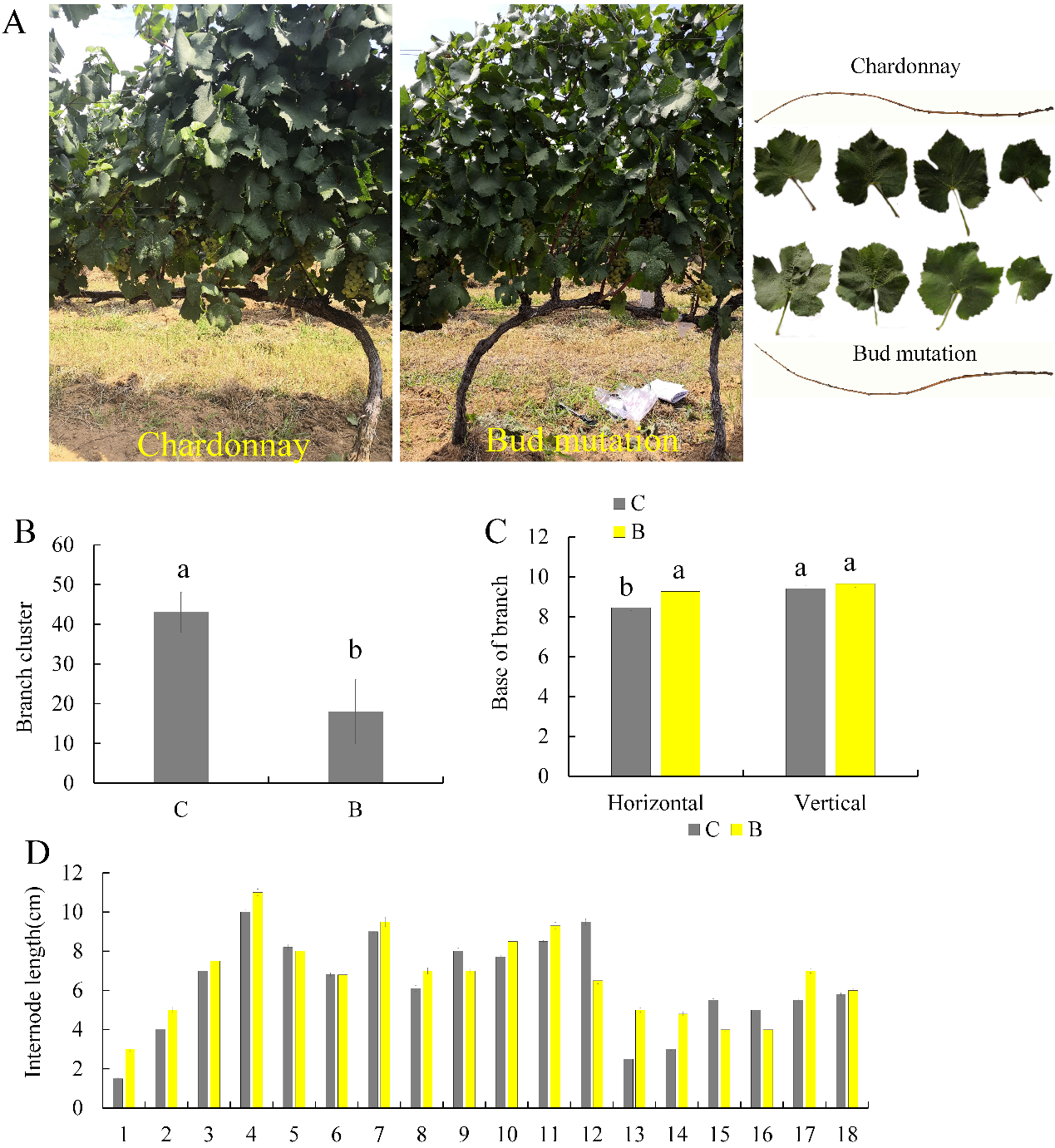


**Figure S1** Phenotypic differences between the 8-old trees of Chardonnay and Bud mutation. (A) Tree and leaves of both cultivars. (B) Branch cluster. (C) The horizontal and vertical of base of branch. (D) The internode length. Vertical bars represented standard deviations (SD) of means (n = 3). Different letters indicated a statistical difference at p < 0.05 as determined by Duncan’s multiple range test.


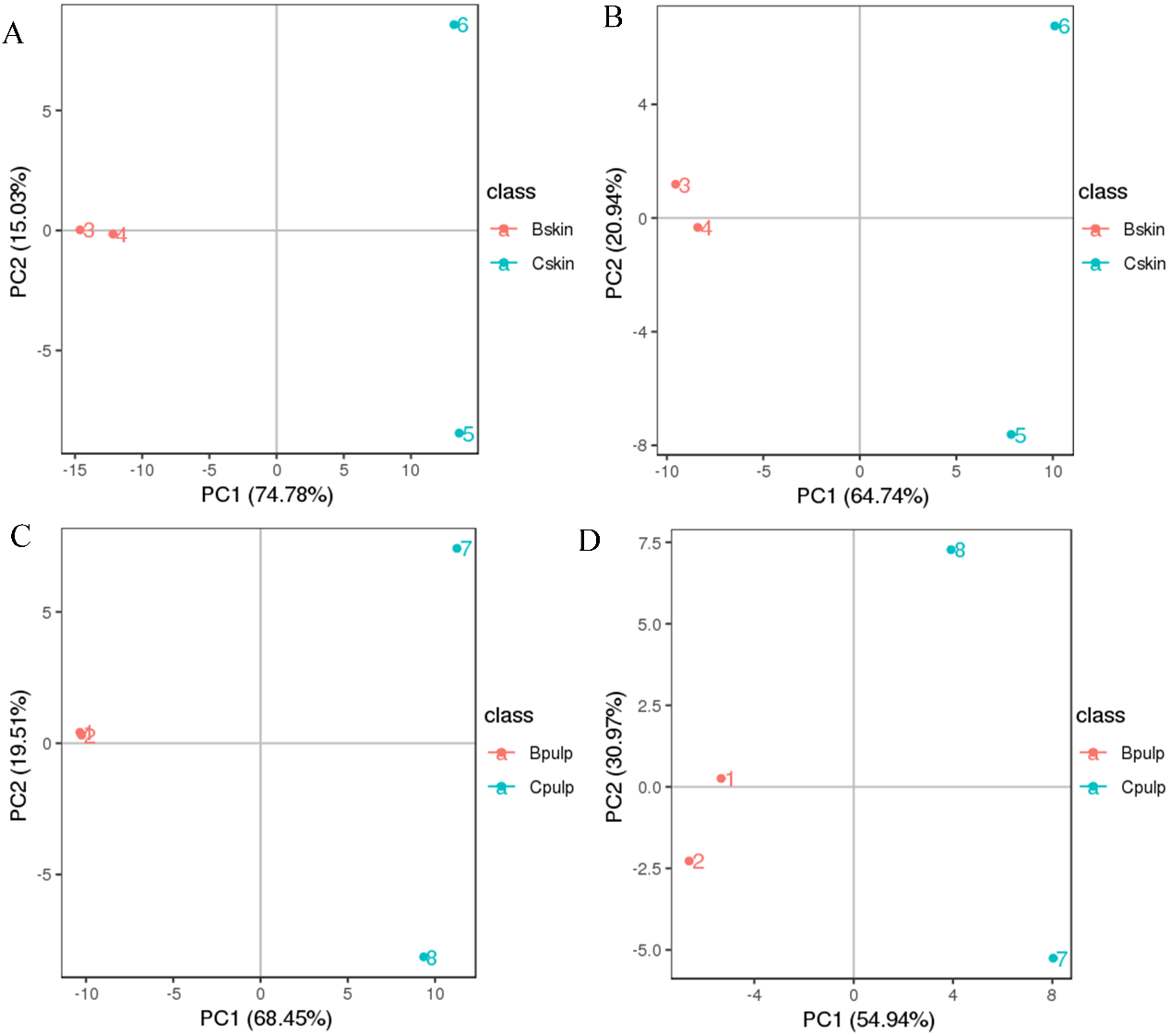


**Figure S2** Principle component analysis (PCA) of metabolites in skin (A,B) and pulp (C,D) of Chardonnay and Bud mutation using two ion mode including positive (A,C) and negative (B,D).


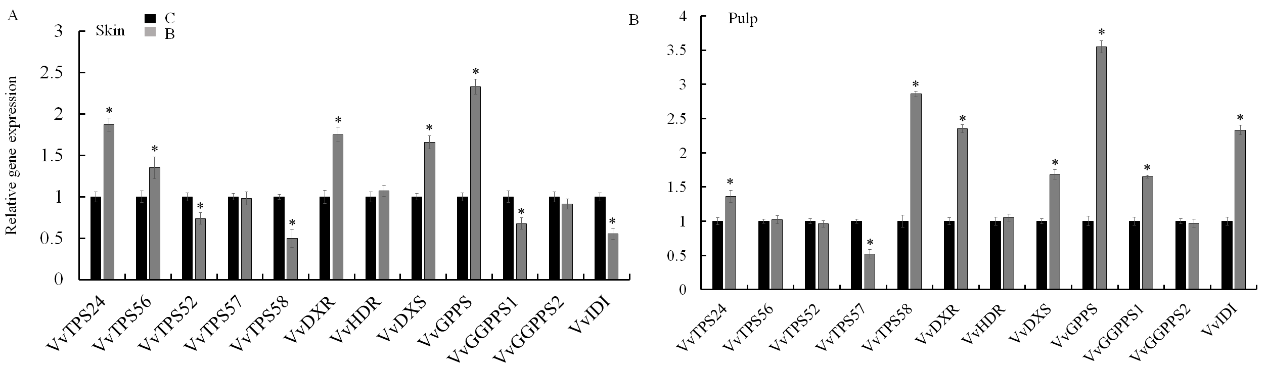


**Figure S3** Genes expression involved in the pathway of terpene metabolism in skin and pulp of Chardonnay (C) and Bud mutations (B) cultivars. * indicates the significance level in P≤ 0.05. C, Chardonnay; B, Bud mutation


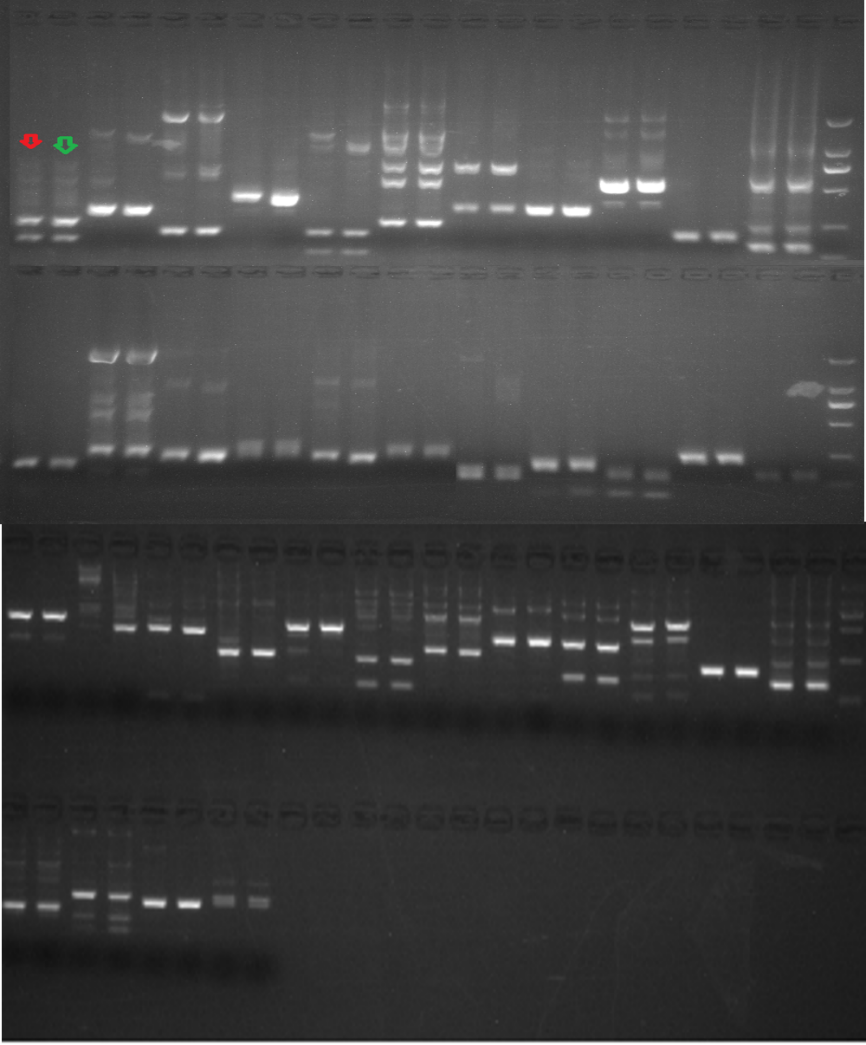


**Figure S4 Chardonnay and Bud mutation have similar genetic background.** 38 pair primers were used to detect the polymorphisms of DNA products of Chardonnay and Bud mutation. According to the map, the results showed that the allele patterns amplified by the two materials were identical, indicating that the genetic background of Chardonnay and Bud mutation was very similar. Red arrow was Chardonnay, green arrow was Bud mutation.
